# Safe storage of pesticides in Sri Lanka – Identifying important design features influencing community acceptance and use of safe storage devices

**DOI:** 10.1186/1471-2458-8-276

**Published:** 2008-08-05

**Authors:** Manjula Weerasinghe, Ravi Pieris, Michael Eddleston, Wim van der Hoek, Andrew Dawson, Flemming Konradsen

**Affiliations:** 1 South Asian Clinical Toxicology Research Collaboration, Faculty of Medicine, University of Peradeniya, Peradeniya, Sri Lanka; 2 Department of International Health, Immunology and Microbiology, University of Copenhagen, Denmark; 3 Scottish Poisons Information Bureau, Royal Infirmary of Edinburgh, Edinburgh, UK; 4 School of Medicine and Public Health, University of Newcastle, Australia

## Abstract

**Background:**

Self-poisoning with pesticides is the cause of an estimated 300,000 deaths annually in rural Asia. The great majority of these deaths are from impulsive acts of self-harm using pesticides that are readily available in the home. The secure storage of pesticides under lock has been emphasized as a possible answer to the problem. This aspect, however, has been poorly researched. In this paper, we report on the design and use, in rural Sri Lanka, of a variety of different lockable storage devices.

**Methods:**

Following a baseline survey of pesticide storage practices, randomly selected households received a pesticide safe storage device. The study was conducted in two phases. In the first phase a total of 200 households in two villages were provided with in-house safe storage devices and two follow-up surveys were conducted seven and 24 months after distribution. The results of the seven month post-distribution survey have already been published. In the second phase, a further 168 households were selected in two additional villages and given a choice between an in-house and an in-field storage device and a follow-up survey conducted seven months after distribution. Both follow-up surveys aimed to assess the use of the device, obtain detailed user feedback on the different storage designs, and to identify problems faced with safeguarding the key. Twelve focus group discussions were held with representatives of households that received a storage device to derive from the community qualitative feedback on the design requirements for such devices.

**Results:**

One hundred and sixty one of the 200 households selected during the first phase were using pesticides at the time of the follow-up survey, 24 months after distribution. Of these 161 households 89 (55%) had the pesticides stored and locked in the provided device. Among the 168 households that were given a choice between an in-house and an in-field storage device 156 used pesticides at the time of survey and of these 103 (66%) selected in-field storage devices and 34% chose in-house storage devices. Of the 156 households, 106 (68%) stored all pesticides in a locked storage device at the time of the follow-up survey seven months after distribution. The majority of households that received an in-field storage device chose to install the device within their compound rather than in the field as they were concerned about the possibility of theft. The preferred design of the storage device was influenced by a number of occupational factors such as land size, crop patterns, types and the quantity of pesticides used. The presence of termites, perceived safety, material used to manufacture the device and ease of location influenced their choice. The study revealed that it was difficult to keep the key to the device hidden from children; and that the person in charge of the key would have easy access to the stored poison.

**Conclusion:**

This study confirms the high acceptance of lockable storage devices by the community although the use of the device reduced over time. A large proportion of pesticides stored within the compound after the introduction of the device may have implications for accessibility to pesticides in the domestic environment. The ability of other household members, including children, to easily find the key is also worrying.

## Background

Self-poisoning with agricultural pesticides is now the most common method of suicide worldwide, causing an estimated 300,000 deaths each year in rural Asia alone [1]. The scale of the problem has been well documented from countries with a large agricultural community such as Bangladesh [2], China [3] and India [4]. Research over the past 10 years has shown that the great majority of deaths follow impulsive acts of self-harm and that the ready availability of pesticides in the homes of rural communities plays a key role [5,6]. The safe and secure storage of pesticides under lock has been emphasized as a possible answer to the problem [7].

Unfortunately, published studies on the use of safe storage devices in rural communities have been few. The community acceptance and use of such devices must first be explored before large scale effectiveness studies are initiated.

Our previous reported study conducted by the same research team in two villages in the North Central Province of Sri Lanka revealed that provision of in-house pesticide safe storage devices had high community acceptance and utilization in the short term [8]. Other pilot studies from elsewhere in Sri Lanka have confirmed a high community acceptance of safe storage devices; as a result, some argue for safe storage devices as an effective method for the prevention of self-poisoning episodes [9].

One concern identified following the study conducted in the North Central Province of Sri Lanka [8] was that the introduction of in-house safe storage devices resulted in pesticides, to a large extent, being moved from their previous storage location in fields away from the house into the house, thereby increasing the risk of impulsive ingestion if the boxes are not completely secure. This is a real risk – during the seven month study of the 200 households, boxes were broken into and pesticides ingested on two occasions. Due to the inherent risks of in-house storage, it was felt that it would be preferable to install safe storage devices away from the household. Therefore, it was decided that a new study in the two additional villages should include an assessment of community interest in the use of in-field storage devices making it possible to keep pesticides safe but away from the house.

Although the study in the North Central Province of Sri Lanka [8] found a high acceptance and use of safe storage devices seven months after the introduction of the devices, an assessment of attrition over a longer period was needed. It was therefore decided to undertake a new survey among the same households included in the first study but this time after 24 months to assess long-term storage practices and use of the introduced devices.

Finally, as a follow up to the first study conducted in the North Central Province of Sri Lanka, it was decided to identify how design features of five different safe storage devices developed after consultation with the farming communities and piloted in the study area would influence use and safe keeping of pesticides.

This paper presents the results from the additional survey conducted 24 months after the households in the two first phase villages were provided with in-house pesticide storage devices; and the findings from the households living in the two additional villages selected to be included into the study, and were provided with either an in-house or in-field storage device, to assess additional research questions.

## Methods

### Study area

The study was carried out in the North Central Province of Sri Lanka, in four farming villages established in the 1960s–1970s under the Rajangana and Mahaweli irrigation development programs. Research was initiated in two of the villages in February 2005 as part of a first phase of a study to assess community acceptance and use of pesticide storage devices and an additional two villages were included in the study in March 2006 as a second phase of the same study. Field research was completed in July 2007.

The four villages were selected because they had a relatively high incidence of acute pesticide poisoning cases during 2004 according to the records of local hospitals.

In each village, at least two thirds of the community was involved in farming their own irrigated lands. Their main income was generated from agriculture; secondary income came from off-farm activities, such as employment in the garment industry or through family members working overseas. Generally, farmers cultivate two seasons a year, with rice in the wet season and rice or vegetables in the dry season. At the time of settlement, each farmer was allocated one hectare of irrigated land and 0.2 hectares of homegarden.

### Household selection

For this study a total of 368 households (200 from phase 1 and 168 from phase 2) were randomly selected out of 685 registered farming households in the four study villages. Verbal informed consent was sought from all participants. Three farming households refused to take part and were replaced by three other households.

Of the 368 households, 200 households in two villages were selected during the first phase of the study which focused on the assessment of the short-term utilization of in-house pesticide storage devices; the results from this have been reported elsewhere [8]. In this paper we report on the use of the in-house safe storage devices among these 200 households 24 months after the initial distribution of the storage devices and without any further community promotion of the devices. The remaining 168 households recruited into the study lived in the two additional villages selected during the second phase and the use of storage devices among this group were assessed seven months after the distribution of in-house or in-field storage devices.

The process of selecting households and the introduction of the project to the community followed the same process as previously presented in Konradsen *et al *2007 [8]. Community meetings were conducted twice in each village during February and April 2005 in the two villages selected during the first phase and during March and April 2006 in the two villages included in the second phase.

### Baseline surveys

Immediately before distribution of the devices, using a structured questionnaire the selected households were interviewed to obtain information on agricultural practices, pesticide use, and storage. Opinions and preferences on the design of storage devices were also sought from the household members at that time.

Information on pesticide storage was based on direct observations by the interviewer, who recorded whether pesticides were kept in the home and whether or not they were kept under lock. Information on storage of pesticides in the field was obtained from an adult member of the household involved in farming activities; pesticides stored in agricultural fields were not inspected. The baseline survey was planned to coincide with the early dry season, a period of high application of pesticides.

Two weeks after the baseline survey, households were provided with their choice of device, free of charge, along with a strong padlock and keys. The household members were encouraged to identify a person within the household to carry the key to the padlock or, alternatively, two people to each carry a key for different padlocks on the device. When the households were presented with the storage devices, the members were encouraged to store all pesticides in the locked device at all times before the keys were handed to an adult family member. This brief talk to each household, together with the two community meetings, was the only structured promotion given to each community.

### Follow up surveys

In the two villages from phase 1 of the study a survey was conducted in May 2007, 24 months after the initial distribution of storage device to assess long-term use and to obtain feedback on the design of the storage devices. In the other two villages from phase 2, a survey was conducted 7 month after the distribution, in December 2006, to assess the short-term use of the device, to obtain detailed feedback on the different storage designs, and to identify problems with safeguarding the keys. In addition to the structured questions outlined in the survey form, the visits to the households also provided an opportunity to engage in more open dialogue focusing on pesticide storage and poisoning attempts with the household members and such information was included on the back of the survey forms as case descriptions and qualitative information.

The detailed process of designing and pilot testing the survey forms has been described in Konradsen *et al *2007 [8]. All survey visits to the selected households were conducted by a male research officer with a university degree and experienced in conducting community level field research in the local vernacular. Two different research officers were involved in conducting the field surveys and the results were quality assured together with the other members of the research team. The household interviews took place from early morning to early evening at a time most convenient to the adult household members involved in farming activities.

### Focus group discussions

A total of 12 focus group discussions, three in each of the four villages, were conducted in the local vernacular, either separately with male and female farmers or as mixed groups, with six to ten participants in each group. The discussions took place from February to June 2005 and again from January to May 2007. The participants in a focus group discussion were selected from among the households that had received a safe storage device; each discussion represented members of different sections of a village recruited into the group following visits by one of the research team members to the individual houses. The discussions took place at the village temple or another location where the meeting could not be interrupted. The issues discussed included the community's perception of health risks of pesticides, perceived need for improved safe storage of pesticides, design requirements for safe storage devices, how best to promote the use of the devices, and how to keep the key to the storage device away from vulnerable individuals. The discussions allowed for differences among gender and age groups to be highlighted. Two researchers were present at all focus groups discussions and information recorded in notebooks.

### Development of safe storage devices

During the introductory meeting with the community the farming households were invited to provide input to the design requirements for a safe storage device and prototypes were demonstrated to farmers during the second community meeting. Prototypes were further developed through interaction with local craftsmen.

**Figure 1:**
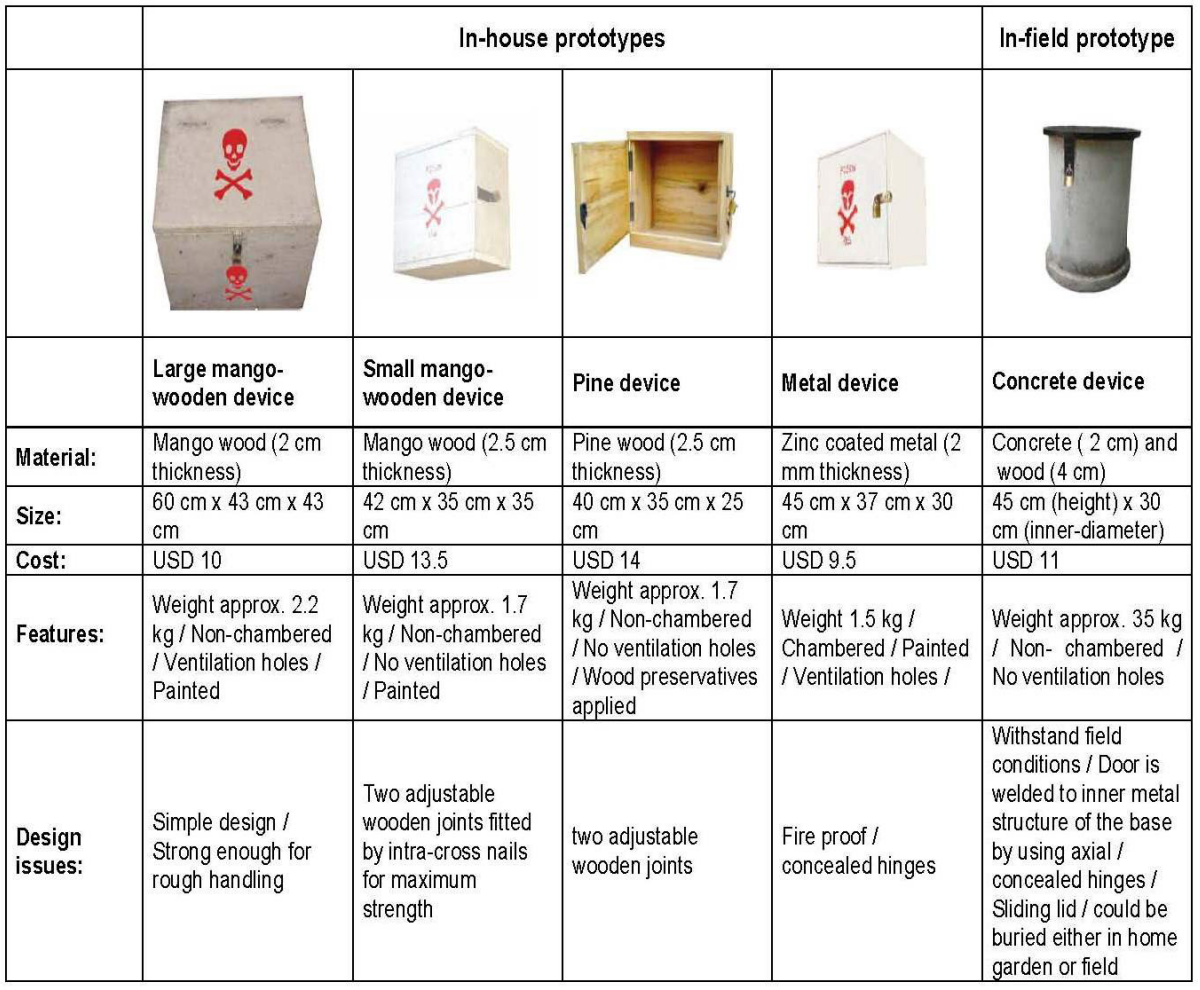
Different prototypes.

Based on farmers' preferences, five prototypes were developed: four in-house devices and one in-field device (Figure 1). The cost of the devices mentioned in Figure 1 was comparable to the price of a container of insecticide or herbicide at one of the private agro-chemical outlets in the study area ranging from 4 USD to 15 USD.

**Figure 2:**
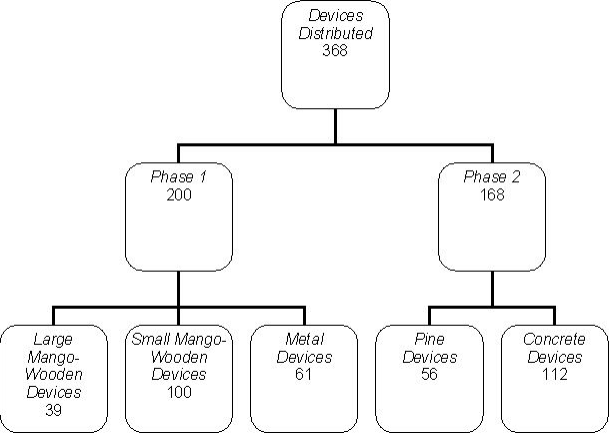
Flow chart showing different device types distributed in phase 1 and phase 2.

Only one device was made available to each household. In phase 1, the community could choose between a large or small mango-wood in-house device and a metal in-house device. In phase 2, the communities were given a choice of either a pinewood in-house device or an in-field concrete device. The number of devices distributed in two phases shows in Figure 2.

### Ethics

Approval for the collection of secondary information from health facilities on reported acute pesticide poisoning episodes by village was received from the University of Peradeniya and Sri Lankan Medical Association Ethics Committees and from the Provincial level of the Ministry of Health. However, the information did not allow for the generation of incidence information at baseline. Verbal consent was sought from all households by the field research team and it was made clear to the families that they were under no compulsion to use the device and could withdraw from the study at any time. Also, during follow-up surveys interviews about the utilisation and acceptability of the device only proceeded after respondents had given verbal consent.

## Results

### Pesticide storage practices

In phase 1 out of 200 households from the two villages where only in-house devices were issued, 29 did not use pesticides at the time of survey 24 months after the distribution of the devices and there were no people present in 10. These 39 households were excluded from the analysis. Of the remaining 161 households, 89 (55%) had all their pesticides stored and locked in the provided device. Another 38 households had placed part of the pesticides in the locked storage device, whilst the balance had been stored elsewhere in the homestead or in the field. This could be compared to the survey that was conducted seven months after the distribution of the storage devices among the same households, when 82% (n = 172) had all their pesticides stored in the locked storage device [8].

Among the 168 households in phase 2, 156 households used pesticides at the time of the follow up survey seven month later, and were available for interview. Of these 156 households, 103 had selected an in-field storage device and 53 an in-house storage device. In the 156 households surveyed at 7 month follow up, 15 (10%) stored pesticides in the field, 81 (52%) in the homegarden and 60 (38%) kept pesticides in the home. Among the 156 households, 106 (68%) stored all pesticides in a locked storage device.

Table 1 shows the baseline and 7 month follow up data of the pesticide storage practices in phase 2 households. Overall, from baseline to 7-months follow-up, the storage of pesticides in the field was reduced from 33% to 10%. The number of households among the 156 that kept pesticides locked away increased from 5% to 68%.

**Table 1:** Comparing storage of pesticides among 156 farming households in Sri Lanka at baseline and seven months after distribution of safe storage devices.

Storage practices*	**Baseline survey**	**Follow up Survey**
Pesticides in house – unlocked	64 (41%)	21 (13%)
Pesticides in house – locked	8 (5%)	39 (25%)
Pesticides in garden – unlocked	33 (21%)	24 (15%)
Pesticides in garden – locked	0	57 (37%)
Pesticides in field – unlocked	51 (33%)	5 (3%)
Pesticides in field – locked	0	10 (6%)

Total	156(100%)	156(100%)

The 156 households could choose between an in-house storage device or an in-field storage device.

* based on observations and interviews with the adult household members

Of the 103 phase 2 households with an in-field storage device, 35 kept the device unlocked seven months after distribution (Table 2). The reason given by 22 of these 35 households was that the padlock was damaged by its frequent contact with soil and water. Among the 328 households combined from phase 1 and phase 2 that used pesticides at 7-months follow-up, the proportion of in-field devices that was unlocked was much greater than for the in-house devices (χ2 = 6.96, P < 0.01). In phase 1 the most significant reduction in locked storage devices between the seven and 24 month follow-up was for the metal devices. However, it was not possible to identify any specific reason for not locking these boxes.

**Table 2:** Utilization of different safe storage devices among 368 (phase 1 and phase 2) farming households in Sri Lanka.

**Type of device**	**No. of households with device**	**No. of households using pesticides at the time of survey**		No. of households with locked devices*		**No. of households with unlocked devices**	
				
		After 7 months	After 24 months	After 7 months	After 24 months	After 7 months	After 24 months
Large device made of mango wood	39	32**	32	25** (78%)	20 (63%)	7** (22%)	12 (37%)
Small device made of mango wood	100	84**	73	70** (83%)	42 (57%)	14** (17%)	31 (43%)
Device made of pinewood	56	53	***	38 (72%)	***	15 (28%)	***
Device made of metal	61	56**	56	46** (82%)	27 (48%)	10** (18%)	29 (52%)
Device made of concrete	112	103	***	68 (66%)	***	35 (34%)	***

Only one storage device was made available to each household.

*Based on inspections made by research team

**Information obtained from Konradsen et al 2007 [8]

***Survey not done in phase 2

### Community preference on parameters for a storage device

#### Site of storage

##### A. In-house storage

During the baseline survey it was observed that those who were concerned about theft of or the effect of the weather on the pesticides selected the in-house storage device. The majority of those who chose the in-house device preferred the version that could be hung from a wall, since it was out of reach of children. However, some households chose the non-hanging version since the mud wall structures in their homes could not support the hanging device.

During the focus group discussions, some male farmers stated that they preferred the in-house storage devices because storage was easier and since the stored pesticides were protected from the sun labels on the containers remained readable and its shell-life was prolonged.

During the focus group discussions it was revealed that if the device was locked at all times, storing pesticides in the home was seen as effective in preventing suicide attempts.

During the focus group discussions and the survey, the female respondents found that the in-house device hanging from an outside wall of the house would be visible to all and make it very easy for the general community to locate; and if the house was empty an individual wishing to consume pesticides could do so easily if the box was not locked at all times. Two third of the participants in these discussions stated that the 'poison' logo should be printed on the outside. Some, especially the users of in-house storage devices, felt that the large 'poison' logo was not a nice symbol to have hanging from the wall.

##### B. In-field storage

The male farmers spoke of the advantages of in-field storage, either with or without a storage device. They felt that having pesticides in the field during the spraying season facilitated their work in the field and that storage in the field would reduce the risk of self-poisoning. In one focus group discussion a male farmer summarized the discussion by stating that "... storage in the field is safer for people considering self-poisoning because most conflicts that lead to self-harm attempts occur in the home environment and if pesticides are stored at home there will always be a risk" and he continued "...unlike in-house storage, in-field storage is safer, even for the custodian of the key if the fields are located a kilometer or two away from the home."

During a follow up household visit, a young male farmer emphasized the importance of the in-field storage from his own experience. He explained that because of domestic-conflicts among the close relatives in the house he had thoughts of committing suicide by ingesting pesticides on three or four occasions but since they usually stored pesticides in the field, about two kilometers away from the house, he had changed his mind before getting access to the chemicals.

The female farmers explained that they appreciated the out-of house storage since this created a healthier environment within the homestead, including the reduced smell of pesticides in the house and accidental poisoning of their children.

During the follow up survey the main argument brought forward for neither selecting nor installing an in-field storage device was the lack of land security. Farmers who did not own land cultivated different small plots of land or shifted among different plots from year to year. These farmers preferred to hide the pesticides in the field they were currently cultivating or install a storage device at home.

##### C. Homegarden storage

Only 11 of the 112 households that selected an in-field storage device actually installed the device in the field (100 meters to 2 kilometers away from the house). The rest installed the device in the home garden (20 to 100 meters away) because they did not own the land and had concerns about theft. Prior to the introduction of the storage device, farmers hid the pesticides in the field and they felt that the device could be located easily and the wooden lid broken into with an agricultural tool. During the follow up survey household members explained that if the cement storage device was installed in the homegarden it would prevent theft, make it possible to store pesticides out of the home and prevent general contamination of the home environment and children accessing them. In focus group discussions household members stated that if a person attempted to break open the in-field storage device located in the homegarden, it would draw the attention of other community members.

Common problems of the in-field storage device were identified at the follow up survey. These included corroded padlocks, broken hinges, water leakage through the wooden lid and high moisture build up inside the device during the rainy season. It was also felt that the device should be able to withstand small fires since farmers often set fire to the fields soon after harvesting and that the device should be painted in a dark shade so that it could not be spotted when buried.

#### Material for in-house devices

The 61 households that selected the metal device did so since metal was perceived to be a strong and durable material. However, during the follow up survey many farmers stated that the device corroded even though it was zinc coated and painted. During the inspection of the boxes it was found that the hinges and staples were corroded. The farmers also found that the pesticide bottles made of glass could break with the rough handling of the metal boxes and feared that there would be a chemical reaction to the metal.

Those who received the wooden device highlighted its durability and easy repair and did not have any complaints. The major disadvantage of the devices made of mango wood was its vulnerability to termite attacks in spite of being painted while the devices made of pinewood were less prone to termite attacks.

The farmers who chose the concrete device, which was designed to be buried, emphasized its durability and the fact that it would be difficult to access by those seeking to harm themselves with pesticides. The weakness of the concrete design was perceived to be the lid that could be broken open. A group of farmers stated that the concrete devices were very heavy, making it difficult to move around.

After using the storage devices for several months those who had chosen the devices made of wood and concrete perceived the locked devices to be a deterrent to intentional pesticide self-harm, while those who had chosen the device made of metal claimed that the metal could easily be bent and the pesticides accessed. In the focus group discussions, the female members highlighted the need for selecting a design that would withstand attempts of opening by their husbands when drunk. For this reason the women preferred the device made of concrete.

#### Size of device

The majority of farmers who cultivate paddy in one hectare of land were satisfied with the size (45 cm (length) × 35 cm (width) × 25 cm (height)) of the "box shaped" device made of pinewood. Farmers cultivating less than two hectares were satisfied with the size (45 cm (height) × 30 cm (diameter)) of the "cylindrical shaped" in-field storage device too since both devices had sufficient space to store one four-liter container, four to six 400 ml bottles and one to two kilograms of pesticide powder. Farmers who cultivated more than two hectares were dissatisfied with the size of the manufactured prototypes. It was observed that farmers preferred to have some space in the device rather than pack it fully. We observed that a small percentage of farmers stored other items such as parts of sprayers, gloves, vegetable seeds, empty bottles etc. in the device. A small group of farmers who chose the in-house storage device expressed concern about space and asked for a smaller device. The farmers who used the in-field device stated that the device should be large and heavy to prevent it from being robbed.

#### Difficulties in keeping the box locked

The follow up survey revealed that in two third of the households the most senior male was in charge of the key to the storage device; in fifteen percent of the households it was an adult female; and in the remaining cases both adult male and female members of the household were responsible. Some farmers claimed that during the cultivating season they frequently used the device and it was difficult to keep opening and locking the device several times per day. As such, they preferred to have a different type of locking system, such as a self-locking device. Some also suggested that regular access to the hidden key increased the chances of the hiding place being spotted by the other family members. The other major issue was the handling of the key in households where more than one person was involved in pesticide spraying. Although farmers were encouraged to use an additional padlock in critical situations, only seven households during the follow up survey were found to have opted to use two padlocks. The other problems with padlocks, identified during the follow up survey, included its corrosion and the loss of the key.

Hiding the key from other family members was still a big challenge to farmers. In the follow up survey among the households that had the storage device for seven months a first visit was made to 42 households where the parents were not at home. The survey team found that in 57% of these households the children could find the key within minutes although they were not responsible for the key. Many respondents mentioned that it was difficult to hide the key in their small houses and some farmers did not believe in the need for hiding the key since they were of the opinion that poisoning would not occur in their household. However, they still wanted to prevent access to pesticides if small children were living in the house.

#### Pesticide poisoning attempts

Although the study was not designed to measure impact of the intervention on pesticide poisoning cases and deaths, all such cases in the study villages that came to the attention of the research team were recorded. Over the 24 month period, 12 severe acute pesticide poisoning cases, including four deaths, were reported in the two villages where only the in-house devices had been distributed. The total population in these villages at the start of the study was 1900, including both individuals living in intervention and non intervention households. Among the 12 pesticide self-poisoning cases registered seven were from families that had received a device. There were two deaths among these seven cases and both individuals were in possession of the key to the device. In five cases the individuals had attempted to force open the storage device and only one succeeded but survived the attempt. No accidental pesticide poisoning cases or severe occupational pesticide poisoning cases were reported.

In the villages where the in-field devices were distributed, with a total population of 2175, two acute pesticide self-poisoning cases were reported in the seven months of observation. However, they did not have storage devices. One used a pesticide that he kept in his house and the other obtained the pesticide from a nearby outlet.

## Discussion

This study confirms the high acceptance of lockable storage devices by the community and the increase in safe storage after the introduction of the devices. However, while appropriate usage was relatively well sustained over time, it was far from universal, with only 55% of the households storing pesticides in a locked storage device after two years; a significant reduction from the 82% found at the survey seven months after distributing the storage devices. Also, the study found that the provision of devices influenced the pattern of storage from the field to within the home compound with a potential of increasing the risk to the household members if the device is not locked at all times. The reduction in usage over time raises the possibility that a continuing education campaign directed at adults and children may improve usage just as it improves compliance with many other health interventions.

The ability of other household members (children) to find the key is also worrying. In addition, the person in charge of the key is highly vulnerable as this person will have easy access to the pesticides, highlighted by the fact that the two deaths from self-pesticide poisoning recorded from the targeted households in this study were persons who had been in charge of the key. Overall, the study supports the need for a large scale study to assess the impact of the mass distribution of pesticide safe storage devices before it can be recommended as a general policy [10].

The preferred design was influenced by a number of occupational factors such as land size, crop patterns, types and the quantity of pesticides used and it was clear that not one single design would suit all farmers. Also, environmental factors such as termites and house construction influences the material used for the manufacture of the device and where it could be located. Aesthetic issues such as colour, logos etc., could also influence placement.

While the high utilisation of in-field devices within the household compound seemed initially surprising to the investigators it was congruent with many of the preferences expressed in the focus group discussions. The improvements suggested by the documented deficits in the current design include improved water impermeability, termite resistance and improved locking. Interestingly, there were no attempts of poisoning in households using in-field devices even though these were the lowest percentage of locked devices.

Households that preferred the in house devices often claimed that they were at low risk of poisoning, had no children or rented their agricultural land. In such households it appears important to address the locking system to be used, such as self-locking locks or combination locks as a solution to key management.

This study may have been strengthened if an increased number of cross sectional surveys had been conducted to assess user patterns of the storage devices. However, the study team decided to limit the number of surveys to avoid influencing the pattern of use by acting as an organization promoting the devices. In the assessments covering only seven months it is possible that the seasonal differences may have influenced how the farmers viewed specific design features of the devices or the cultivation patterns may have influenced use in ways that could only be researched if the study period included the two cultivations seasons practiced in the area. Finally, it is possible that the study results focusing on end user aspects may have been influenced by the fact that all members of the research team were males.

## Conclusion

A variety of safe storage devices is needed to ensure a "best fit" for each household. Arguably those households who cannot sustain safe storage may be at the greatest risk of self poisoning and the challenge remains to ensure sustained appropriate usage by recurrent promotion and incorporation into community expectations

## Competing interests

The authors declare that they have no competing interests.

## Authors' contributions

MW, RP and FK were involved in designing the study and the format for data collection. MW and RP were responsible for field data collection and data entry. FK and WvdH were responsible for data analysis and produced the first draft version of the paper. ME and AHD contributed with significant input to the revisions of the manuscript and provided significant intellectual content throughout the project. All authors approved and agreed to the final manuscript.

## Pre-publication history

The pre-publication history for this paper can be accessed here:


